# Cancer Immune Evasion Through Loss of MHC Class I Antigen Presentation

**DOI:** 10.3389/fimmu.2021.636568

**Published:** 2021-03-09

**Authors:** Karthik Dhatchinamoorthy, Jeff D. Colbert, Kenneth L. Rock

**Affiliations:** Department of Pathology, UMass Medical School, Worcester, MA, United States

**Keywords:** antigen presentation, cancer immune evasion, MHC I antigen presentation, interferon, TAP1, Tapasin, epigenetic regulation

## Abstract

Major histocompatibility class I (MHC I) molecules bind peptides derived from a cell's expressed genes and then transport and display this antigenic information on the cell surface. This allows CD8 T cells to identify pathological cells that are synthesizing abnormal proteins, such as cancers that are expressing mutated proteins. In order for many cancers to arise and progress, they need to evolve mechanisms to avoid elimination by CD8 T cells. MHC I molecules are not essential for cell survival and therefore one mechanism by which cancers can evade immune control is by losing MHC I antigen presentation machinery (APM). Not only will this impair the ability of natural immune responses to control cancers, but also frustrate immunotherapies that work by re-invigorating anti-tumor CD8 T cells, such as checkpoint blockade. Here we review the evidence that loss of MHC I antigen presentation is a frequent occurrence in many cancers. We discuss new insights into some common underlying mechanisms through which some cancers inactivate the MHC I pathway and consider some possible strategies to overcome this limitation in ways that could restore immune control of tumors and improve immunotherapy.

## Introduction

Highly immunodeficient mice, which completely lack adaptive immunity, develop high rates of spontaneous and carcinogen-induced cancers (1, 2). Similarly, immunodeficient humans suffer from higher rates of malignancy (3–5). Therefore, the immune system is capable of recognizing and eliminating many cancers before they become clinically evident. Moreover, cancers that are infiltrated with activated T cells often have better prognosis, indicating that the immune system can exert some control on cancers, even after they have become clinically evident (6–15). Further evidence that the immune system has the potential to control and/or eliminate cancers has come from the success of immunotherapies, such as checkpoint blockade. In checkpoint blockade immunotherapy, patients are treated with antibodies that block negative regulatory molecules, such as PD-1/PD-L1 or CTLA4, which normally restrain T cell responses. This kind of therapy can reinvigorate a patient's anti-tumor T cell responses, which then can cause tumors to shrink and even lead to cures in some patients (16, 17). While all these observations show that the immune system has the capacity to fight cancer, the unfortunate fact is that once the majority of cancers have become clinically evident, untreated they almost always continue to progress and a majority fail to respond and/or be eliminated by checkpoint blockade immunotherapy. Therefore, understanding how cancers evade immune control is important for understanding tumor pathogenesis and for devising ways to improve immunotherapy.

**Figure 1:**
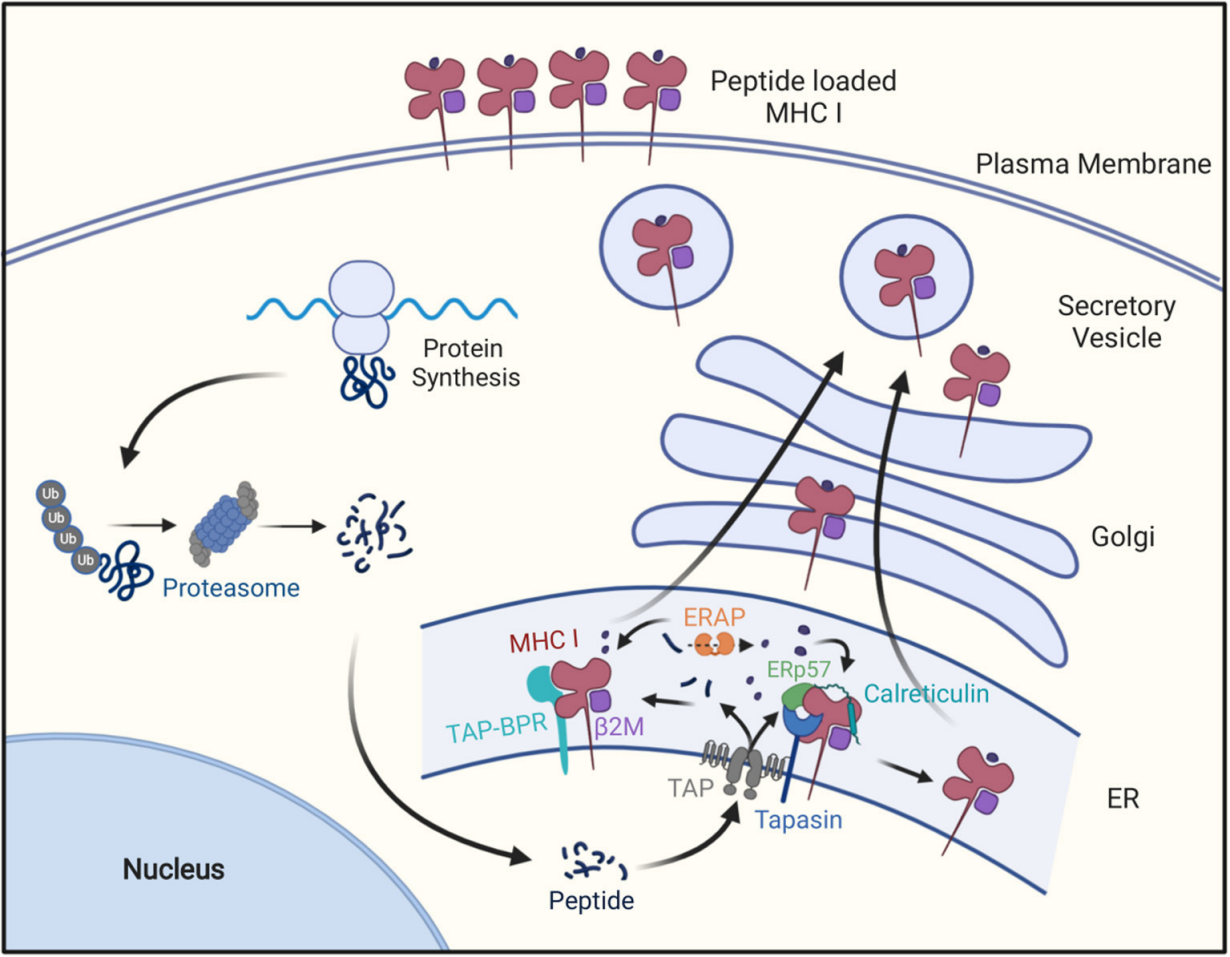
The MHC class I antigen presentation pathway. Cellular proteins are hydrolyzed by the ubiquitin-proteosome pathway into oligopeptides, which are subsequently transported into endoplasmic reticulum through the TAP transporter. In the ER these peptides may be further trimmed by ERAP1 and then peptides of the right length and sequence bind to MHC I molecules with the help of Tapasin in a peptide-loading complex containing Tapasin, TAP, calreticulim, and ERP57, or the with the help of TAPBPR. After MHCI molecules bind peptide, they are transported to the cell surface for display to CD8^+^ T cells.

While there are several immune effector mechanisms that can damage tumors, the most important ones are carried out by CD8 T cells. This has been shown [e.g., in experiments where tumor rejection was inhibited in mice that were depleted of CD8 T cells (18, 19)]. Similar principles are thought to apply in humans as shown [e.g., by the observations that the presence of activated CD8 T cells in cancers are associated with improved survival (20) and adoptive immunotherapy with T cells engineered to express TCRs from tumor-reactive CD8 T cells can lead to cancer regression (21)]. Tumor-reactive CD8 T cells identify cancers by recognizing peptide-MHC I complexes that are generated through the MHC I antigen presentation pathway (Figure 1 and below). Upon recognizing a cancer, CD8 T cells go on to kill these cells *via* perforin or FAS-dependent pathways and also can injure tumors by inciting inflammation. Such mechanisms are important in controlling cancer as shown [e.g., by the finding that higher frequencies of cancers develop in perforin-null or FAS-deficient mice (22, 23) compared to their wild type counterparts and potentially also in perforin-deficient humans (24)].

In order to progress, cancers need to circumvent immune control. This was nicely illustrated by a study of carcinogen-induced cancers that arose in immunodeficient vs. immunosufficient mice. Cancers from immunodeficient mice grew when transplanted into other immunodeficient mice. However, these same cancers were generally rejected in wild type mice, showing that they were inherently immunogenic (1). In contrast, tumors that arose in wild type mice would often grow when transplanted into other wild type mice (1). These findings indicated that tumors that arose in the presence of the intact immune system in wild type mice evolved in ways that allowed them to evade immune elimination (2). This evolution of cancers under selection pressure from CD8 T cells has been referred to as “immunoediting” (2, 25).

Cancers are often genetically unstable and can lose expression of non-essential molecules through gene loss or epigenetic silencing. MHC I molecules and most of the other molecules of the MHC I antigen presentation pathway are not essential for cell viability or growth (see below). Consequently, cancers can down-regulate or lose MHC I antigen presentation, and thereby become less stimulatory or even invisible to CD8 T cells, without impairing their ability to grow and metastasize. In this article we will review the incidence, underlying mechanism, and therapeutic implications of loss of MHC I in cancers. Except where noted, this review primarily focuses on human cancers, because of their clinical importance. It should be noted that cancers can also evade immune elimination by expressing “non-classical” MHC class Ib molecules, HLA-E and HLA-G (26–28). However, since this immune evasion mechanism is not due to a loss of antigen presentation by “classical” MHC class Ia molecules, but rather through engagement of inhibitory receptors on T lymphocytes and other immune cells (26–28), this subject is not covered in this review, except as it relates to how MHC I low cancers may evade NK cell recognition. Similarly, MHC II molecules can play a role in cancer immunity, however, since MHC I and MHC II antigen presentation are separate and non-intersecting pathways, this review does not cover the MHC II pathway in cancer.

## The MHC Class I Pathway of Antigen Presentation

To understand some of the mechanisms by which many cancers evade immune surveillance, it is necessary to first understand the MHC I pathway of antigen presentation (Figure 1). This pathway is the mechanism that allows CD8 T cells to identify cells producing “foreign” proteins, such as ones from viruses in infected cells or mutant genes in cancers. In this pathway, MHC I-presented peptides are generated as part of the normal catabolism of cellular proteins. All endogenously synthesized proteins are continuously degraded into oligopeptides by the ubiquitin-proteasome pathway (29). This catabolic pathway is responsible for making the initial cleavages, and particularly the proper C-terminal cut, needed for the generation of a majority of MHC I-presented peptides (29–32).

There are several forms of proteasomes, known as proteasomes, immunoproteasomes and thymoproteasomes (33). Immunoproteasomes are formed when three alternate versions of proteasome active site subunits are expressed in cells and preferentially incorporate into newly assembling proteasomes in place of the standard active site subunits. Since these alternate active sites have different catalytic properties, immunoproteasomes generate many different (as well as some of the same) peptides as proteasomes and it seems that the ones produced by immunoproteasomes are often better for presentation on MHC I molecules (34, 35). Cells and animals that genetically lack the three immunoproteasome subunits are viable (35).

A fraction of the peptides produced by proteasomes and immunoproteasomes are transferred into the lumen of the endoplasmic reticulum (ER) by a peptide transporter called TAP (36). TAP can transport most, but not all, peptides that are between 9 and 13 residues in length (37–39). TAP is composed of two different subunits (TAP1 and TAP2) and both are needed for transporting peptides (40–43). Upon transport into the lumen of the ER, peptides are in the vicinity of newly assembling MHC I molecules.

The heavy and light [ß2-microglobulin (ß2M)] chains of MHC I molecules are co-translationally transported into the ER where they fold into the MHC I heterodimer. Before binding these complexes are inherently unstable and are stabilized through interactions with chaperones such as calreticulin within a multi-protein complex, called the peptide-loading complex (44, 45). Other components of this complex include the peptide transporter TAP, the oxidoreductase ERP57 and the peptide “editor” Tapasin. Tapasin helps retain peptide-empty MHC I molecules in the ER and also promotes their loading with high affinity peptides (46, 47). There is another peptide-editor called TAPBPR, which is not part of the peptide-loading complex, that also promotes peptide-loading of MHC I molecules (48). Cells and animals that lack Tapasin, ERP57, or TAP are viable (49, 50).

The empty MHC I molecule contains a groove that binds peptides (51, 52). Peptides are bound *via* molecular interactions typically with two of their side chains, some of their main chain atoms and their free N- and C-termini (47). Because the interactions with the peptide's two ends contributes substantially to the affinity with which peptides are bound, most MHC I-bound peptides are of a uniform length, which depending on the particular MHC I molecule is typically 8, 9, or 10 residues (53). Proteasomes and immunoproteasomes make some peptides in this size range, but also many more that are too short or too long for stable binding to MHC I molecules (54). However, the long peptides can be trimmed to the proper size for presentation. Much of the trimming of long peptides occurs in the endoplasmic reticulum by an aminopeptidase called ERAP1 (ERAAP) (55, 56) and in humans also a second related peptidase called ERAP2 (57). ERAP1 is specialized in trimming long peptides to the optimal length for binding MHC I molecules, as it slows or stops trimming most peptide substrates when they are 8–9 residues in length (55). Long peptides can also be trimmed by aminopeptidases in the cytosol and the resulting shorter peptides can be transported by TAP into the ER (58). Similar to TAP and MHC I mentioned above, cells and animals that lack ERAP1 are also viable (59–62).

The peptides produced by these various mechanisms that have the right length and sequences can then bind to the empty MHC I molecules in the ER, often assisted by the peptide-editors Tapasin and TAPBPR. Upon binding peptides MHC I complexes are both stabilized and released from the ER, whereupon they follow the default exocytic pathway to the plasma membrane for display to CD8 T cells. In cells that have defects in making, transporting or MHC I-loading of peptides, most of their MHC I molecules are retained in ER and ultimately degraded, resulting in a paucity of MHC I molecules on the cell surface (29, 32, 63). As will be discussed further below, such defects underlie the MHC I low phenotype in many cancers.

## Regulation of the MHC I Pathway of Antigen Presentation

**Figure 2:**
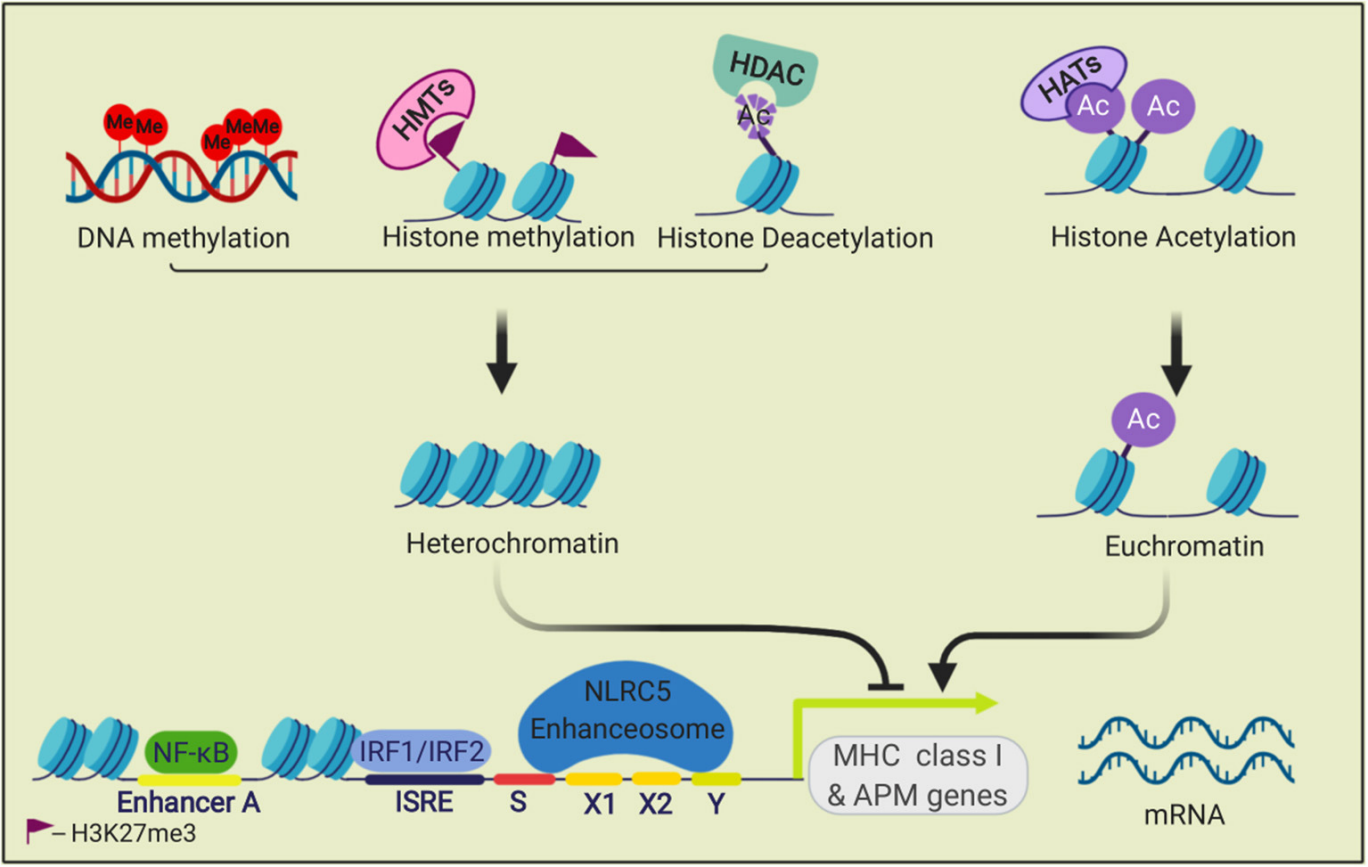
Transcriptional regulation of MHC class I genes. The transcription factors NF-κB, IRF1, and IRF2, and the NLRC5 enhanceosome bind to promoter and enhancer elements in the 5' upstream sequences of MHC I APM genes and drive their transcription. This process is regulated by epigenetic modifications. Methylation of histone (H3K27me3) by histone methyltransferases (HMTs) and DNA methylation repress transcription. Histone acetyltransferases (HATs) acetylate histones, which can open the chromatin for transcription. Histone deacetylases (HDACs) can remove histone acetylation marks and silence transcription.

Regulation of MHC I antigen presentation is also relevant to tumor immune evasion. The expression of most components of the MHC I antigen presentation pathway, including MHC I heavy chains, ß2M, immunoproteasome subunits, TAP, Tapasin and ERAP1, are coordinately regulated. This is because these antigen presentation components all have similar gene control elements in their promoters/enhancers (64, 65) (Figure 2). These elements include sequences that bind the transcription factors NLRC5-enhanceosome, NF-κB, and IRF1/IRF2 (66). Gene silencing or editing experiments have shown that the NLRC5, IRF1, and IRF2 transcription factors are essential for basal and/or interferon (IFN)-induced MHC I expression, but not for cell viability or growth (67–70). Since these are non-essential genes (71, 72), their expression can be lost in cancer cells, as will be discussed below.

**Figure 3:**
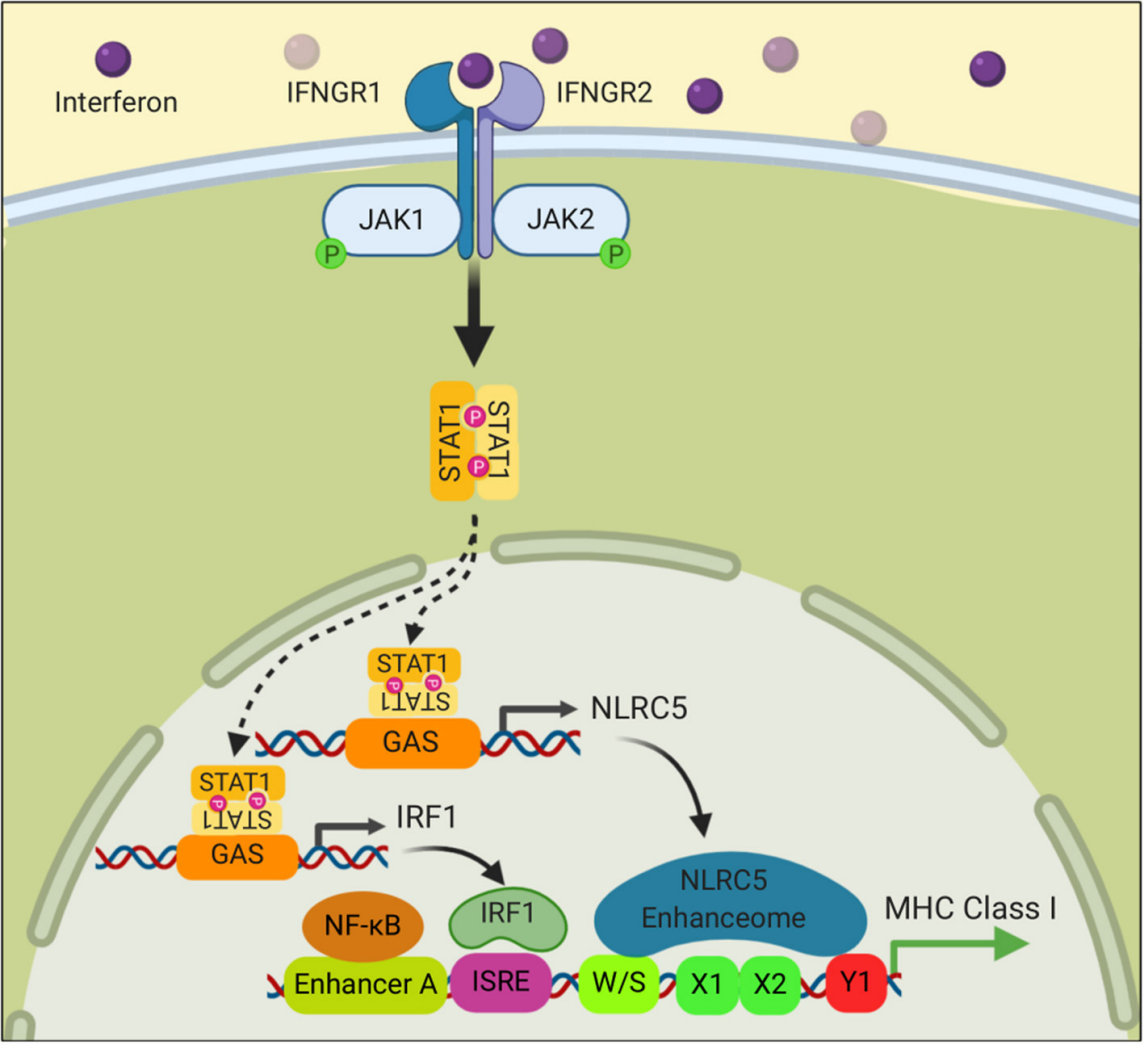
Interferon signaling stimulates the transcription of MHC class I genes. Binding of Interferon to its receptor stimulates phosphorylation of the Janus kinases, JAK1 and JAK2, which in turn phosphorylate STAT1. Phosphorylated STAT1 translocates into the nucleus where it binds to promoter elements of NLRC5 and IRF1 and drives their transcription. NLRC5 and IRF1 then stimulate MHC I gene transcription as described in Figure 2.

Many cells of hematopoietic origin, such as dendritic cells and lymphocytes, constitutively express relatively high amounts of all of the MHC I antigen presentation components and consequently without any stimulation have high levels of MHC I molecules on their cell surface (73, 74). In contrast, under basal conditions, most other cells have lower expression of these components and have less MHC I on the cell surface (74). However, in all cells, the expression of MHC I pathway components and surface MHC I levels are increased upon stimulation with interferons, especially type 2 IFN (IFNγ) (75). In such responses, IFNs upregulate expression of IRF1, STAT1, and NLRC5 (76), which then binds to the promoters of the antigen presenting gene and drives their expression (Figure 3). IFNs are induced in response to infections and T cell responses, this upregulated expression is thought to enhance detection of pathological cells. That this mechanism is important in cancers is suggested from studies that have documented an increased incidence of cancer in mice that have defects in the IFN pathway (77, 78), and that in humans IFNγ-signaling signatures in cancers correlate with response to immunotherapy (79).

## Cancers Antigens and Their Visibility to the Immune System

Through the mechanisms describe above, all cells display on their surface peptides from a majority of the proteins that they are making. This process allows CD8 T cells to identify and eliminate cells that are synthesizing “foreign” or other immunogenic proteins. Foreign (non-self) sequences in cancer may come from endogenous genes harboring mutations, which are often referred to as neoantigens, or in some cases from viral sequences in cancers (e.g., human papilloma viral proteins in human cervical carcinomas) (80).

Mutational burdens vary substantially among cancers. Tumors with higher mutational burdens are theoretically more immunogenic, and there is some evidence to support this concept. Melanomas and non-small cell lung carcinoma (NSCLC) often have high mutational burdens (81) and are considered more immunogenic tumors. There is also a correlation between the number of mutations in cancers and their responses to checkpoint blockade or adoptive T cell immunotherapy (82–84). This has been interpreted to suggest that cancers, which display many immunogenic peptides, will be much more likely to be attacked by CD8 T cells that have been reinvigorated by immunotherapy. In addition to mutated peptides, there are other kinds of immunogenic tumor antigens. For example, anti-cancer CD8 T cells can recognize de-repressed oncofetal antigens, cancer germline antigens, and even normal (non-mutated) cellular antigens, such as tyrosinase in melanomas and melanocytes (85, 86). In this latter case, the responding T cells are autoreactive ones that have escaped normal tolerance mechanisms and are present in the T cell repertoire. However, cancers that lack any immunogenic antigens are ones that can't be controlled by CD8 T cells.

Cancers that are initially immunogenic can lose visibility to CD8 T cells in two general ways. If the immunogenic antigens are non-essential for cell survival, and this is probably true for a majority of tumor antigens, then genetically unstable cancer cells can lose expression of the cancer antigens (87–90). After this occurs, CD8 T cells will be ineffective in controlling the cancer because despite the tumors having plenty of MHC I molecules, the cancer cells have lost all antigenic peptides that CD8 T cells can recognize. This route of immune evasion will be less likely in cancers that express many immunogenic cancer antigens because it would require simultaneous loss of expression of many independent gene products. This may be another reason as to why tumors with high mutational burdens are more susceptible to T cell immunotherapy. The other general way that cancers can lose visibility to CD8 T cells is by down regulating the MHC I antigen presentation pathway. The evidence that this occurs, and its underlying mechanisms and clinical importance are considered in the following sections.

## Cancers Often Lose Expression of MHC I Molecules

**Figure 4:**
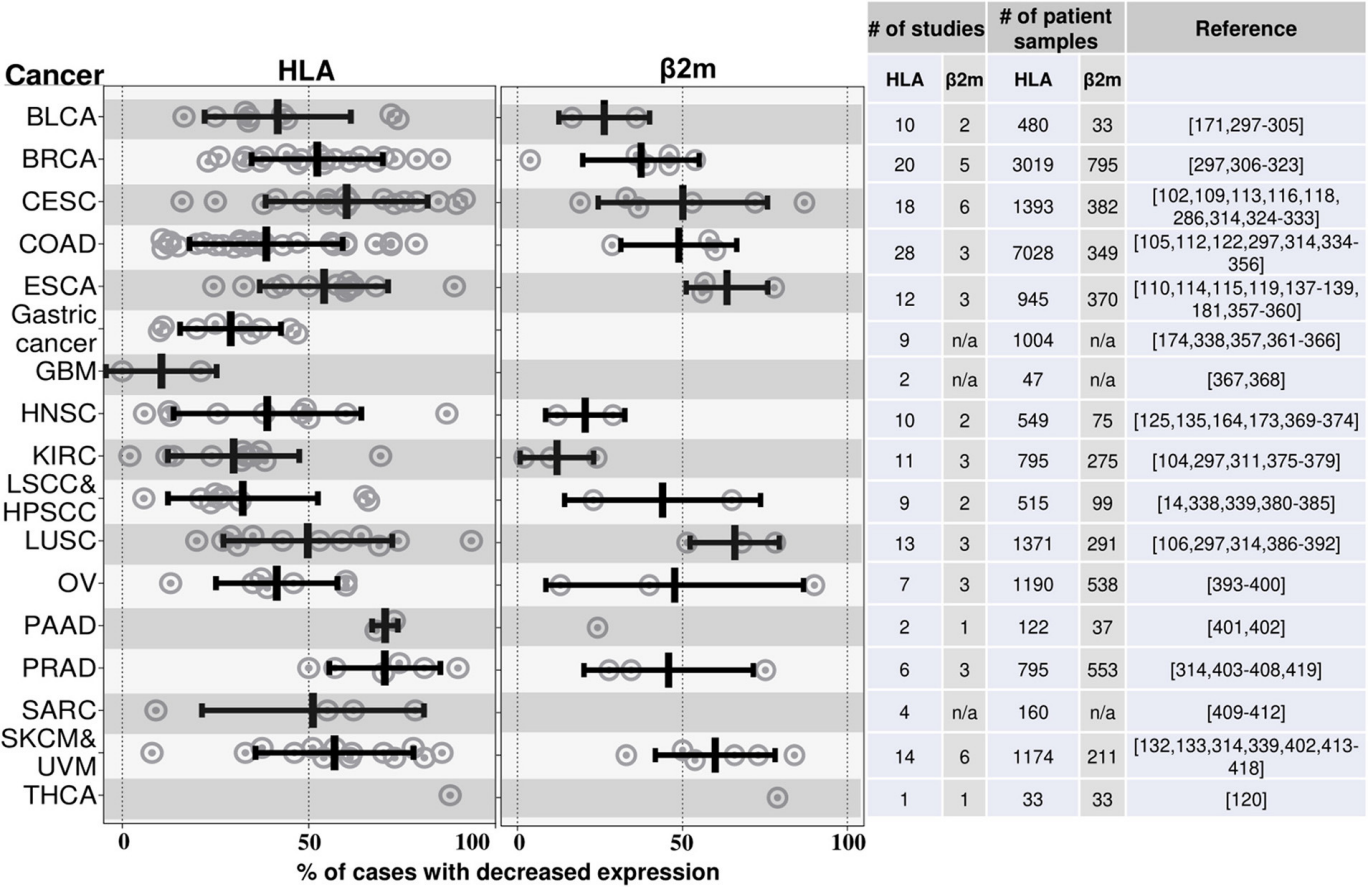
HLA and β2M are frequently downregulated in many different cancers. This graph illustrates the findings from a number of studies that have measured MHC I expression in various cancers by immunohistochemistry. Cancers are annotated by their TCGA abbreviations (see abbreviation list). Each dot represents the percent of cases with loss of MHC I expression in an individual study. The mean % reductions and standard deviations for all of the studies combined are shown by the black bars. The data to the right of the graph shows the number of studies and number of patients samples used to quantify the MHC class I. The studies that were included are shown in the references; this is not an exhaustive list of all such analyses. BLCA, Bladder urothelial carcinoma; BRCA, Breast invasive carcinoma; CESC, Cervical squamous cell carcinoma and endocervical adenocarcinoma; COAD, Colon adenocarcinoma; ESCA, Esophageal carcinoma; GBM, Glioblastoma multiforme; HNSC, Head and neck squamous cell carcinoma; KIRC, Kidney renal clear cell carcinoma; LSCC, Lung squamous cell carcinoma; HPSCC, Hypopharyngeal squamous cell carcinoma; LUSC, Lung squamous cell carcinoma; OV, Ovarian serous cystadenocarcinoma; PAAD, Pancreatic adenocarcinoma; PRAD, Prostate adenocarcinoma; SARC, Sarcoma; SKCM, Skin cutaneous melanoma; UVM, Uveal melanoma; THCA, Thyroid carcinoma; IFN, Interferon; LIHC, Liver hepatocellular carcinoma; NSCLC, Nonsmall cell lung carcinoma.

A large number of many different types of human cancers have been reported to lose expression of MHC I molecules to varying degrees (Figure 4). An MHC-low phenotype has been observed in many of the most frequent human cancers including NSCLC, breast, prostate, colorectal, head and neck squamous cell carcinoma (HNSC), hepatocellular carcinoma, and melanoma. The number of cases that have lost MHC I expression varies for different types of these cancers and between different studies, and ranges from 0 to 93% (Figure 4). Cancers may not be homogeneous and can have variable expression of MHC I among its cells and/or in different regions. In addition, expression may change over time as a cancer progresses and may differ between the primary site and metastases (91–94).

The vast majority of these studies have analyzed MHC I expression in primary patient samples by immunohistochemistry (IHC) using antibodies specific for monomorphic determinants on the heavy chains of classical MHC I molecules (HLA-A, HLA-B, and HLA-C) or for ß2M. Therefore, many cancers have downregulated MHC I antigen presentation broadly. Loss of expression of a single MHC I molecule has also been reported (95, 96). Many studies have reported cancers that are MHC I negative, however because of the limits of sensitivity of IHC, it is possible that some of these cases may still express some MHC I molecules.

As described above, because peptide empty MHC I molecules are unstable without chaperone-binding and retained in the ER, defects almost anywhere in the MHC I pathway (e.g., loss of MHC I heavy chain, ß2-microglobulin, immunoproteasome subunits, TAP, Tapasin, and ERAP1) results in a loss of MHC I molecules from the cell surface. In mouse and human cells, genetic deletion of TAP reduces MHC I levels by 30–70% for most MHC I alleles (40, 97). Similarly, loss of Tapasin decreases MHC I expression by as much as 90% and deletion of immunoproteasome subunits reduces MHC I levels by ~50% (35, 98). MHC I levels are reduced in ERAP1 KO cells by 20–70% (59, 99, 100).

**Figure 5:**
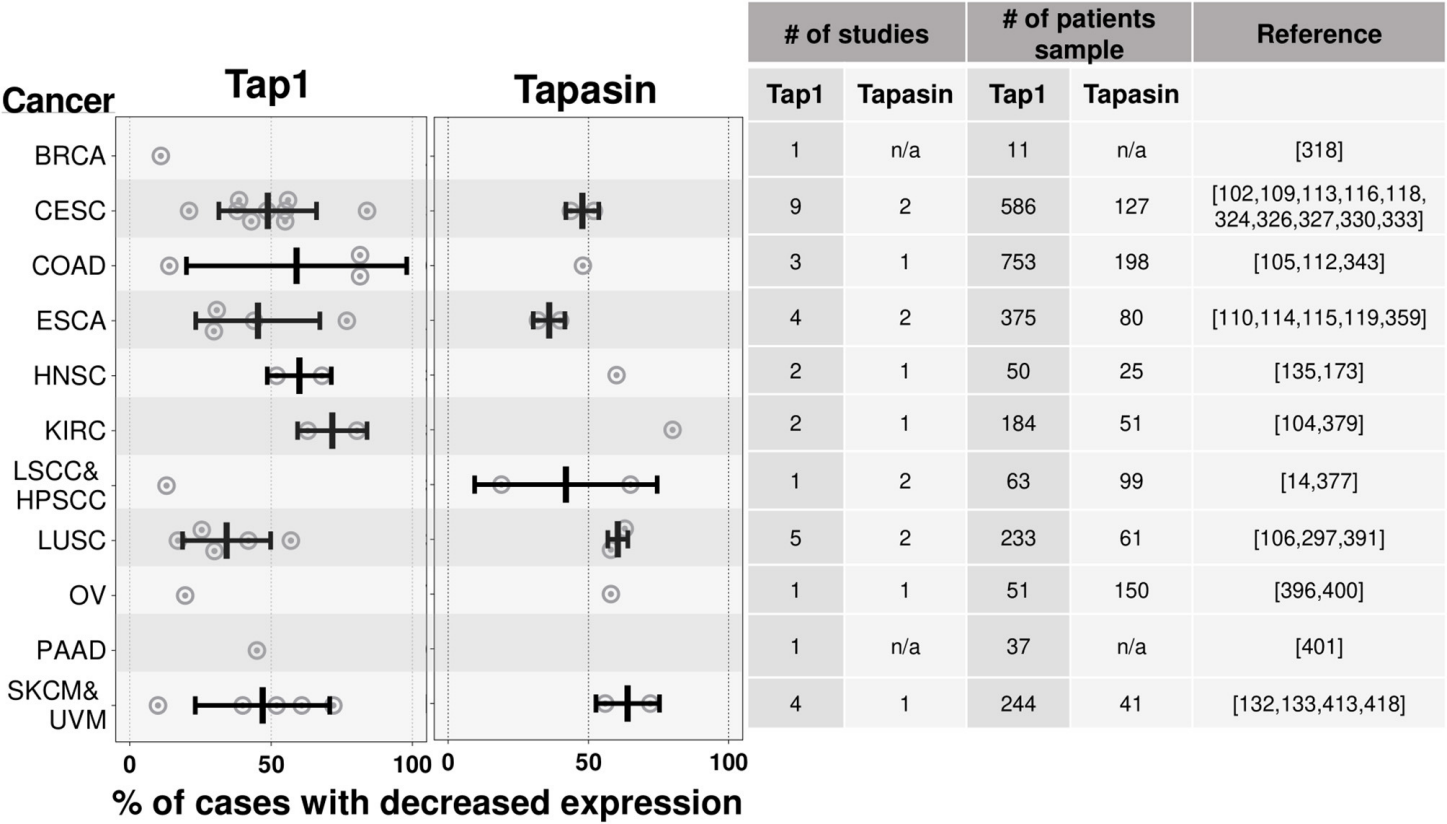
Tap1 and Tapasin are downregulated in many cancer types. Similar to Figure 4, except summarizing results from studies quantifying loss of Tap1 (one of the chains of the TAP transporter) and Tapasin, instead of MHC I. There are fewer studies of the expression of these proteins in cancers relative to the studies of MHC I expression in cancers. n/a-data is not available. Cancer abbreviation are as in Figure 4.

The expression of these antigen presentation pathway components in cancers has been studied (101) but much less extensively than for MHC I and ß2M. Of these components, TAP has been studied most extensively. Loss of TAP expression, ranging from 10 to 80.4%, has been documented in colorectal, renal cell cervical cancers, and melanomas (102–110) (Figure 5). There are more limited studies that have documented loss of expression of Tapasin (111–114), Immunoproteasomes (113, 115), and ERAP1 (116–119) (Figure 5). Individual cancers can lose expression of multiple of these antigen presenting components (see section on transcriptional regulation below) and the net effect of these multiple loses on MHC I expression should be compounded.

## Clinical Importance of Loss of MHC I Expression In Cancers

As discussed above, the loss of MHC I antigen presentation will make cancers less visible to the immune system and this is predicted to impair control of such tumors by CD8 T cells. There are three lines of evidence that support this concept in human cancer patients. First, in some cancers the presence of tumor-infiltrating lymphocytes (TIL), which is often an indication of a host immune response, is positively correlated with MHC I molecule expression on tumor cells. For example, MHC I-low cancers (e.g., breast cancer) contain fewer TIL than their MHC I-high counterparts (10, 11, 120, 121). Since TILs are a positive prognostic feature in many cancers (7–10, 12, 13, 15, 122), the correlation of TIL with MHC I expression is consistent with a role of antigen presentation in immune control of cancers.

A second line of evidence for the clinical significance of MHC I-loss, comes from studies that have correlated MHC I expression with prognosis. In many cancers, including melanoma, glioblastoma, colorectal, bladder, uterine, cervical, head/neck, breast and other cancers, loss of MHC I is associated with worse clinical outcomes (14, 110, 111, 122–140). Since loss of the MHC I antigen presentation pathway does not alter intrinsic cell growth or viability, this correlation is also consistent with a role for antigen presentation in immune control of cancers (128).

The third line of evidence for the clinical importance of MHC I-loss comes from studies of immunotherapy. In several studies, loss of MHC I expression has been correlated with resistance to checkpoint blockade (124, 141–147) and adoptive immunotherapy (148, 149). Moreover, during immunotherapy it was observed that MHC I high metastases were the ones that regressed while MHC low metastases progressed (150). Similarly, defects in IFN-response pathways, which regulate MHC I levels, as described above, are also correlated with resistance to checkpoint immunotherapy (79, 122, 151–153).

These three lines of evidence point to the likely importance of MHC I-loss to clinical outcomes. However, there is a “chicken and egg issue” that should be considered. Since activated CD8 T cells and CD4 Th1 cells produce IFNγ, which can upregulate the MHC I pathway, this raises the question of which of the events came first: High MHC I or the T cell immune response. Similarly, if higher MHC I levels are a consequence of a preexisting T cell responses, then the presence of the responsive T cells could also be the reason that these cases are more responsive to checkpoint blockade immunotherapy. In other words, high MHC I could be an effect rather than a cause of TIL infiltration and the consequent improved clinical responses. While there is undoubtably interplay between MHC I stimulating T cells and T cells stimulating MHC I antigen presentation, the fact is that MHC I is needed to initiate this process (154). Therefore, loss of MHC I antigen presentation is likely causally related to clinical outcomes. In support of this concept, in an experimental human xenograft model, wherein the preexisting T cell repertoire is identical and the only variable is whether a tumor is MHC I high vs. low, loss of MHC I antigen presentation results in resistance to checkpoint blockade (141).

If MHC I expression is a key factor needed for immune control of cancers, as is expected from the underlying science and suggested by the above clinical data, then it is important to understand the underlying mechanisms for MHC I loss. This is of obvious importance for understanding pathogenesis and also for evaluating whether there are ways to potentially restore MHC I expression to improve therapy.

## Loss OF MHC I Expression in Cancers Through Mutation or Deletion of Structural Genes

Many cancers are genetically unstable and can lose gene expression through deletions or mutation of chromosomal sequences (**Figure 7**). Many of the MHC I antigen presenting components (e.g., MHC I heavy chains, TAP, Tapasin, immunoproteasome subunits) are encoded in the MHC on Chromosome 6. Cells that sustain homozygous deletion of large regions of the MHC region are viable and proliferate (155–157) and therefore such chromosomal deletions are permissive in cancers, as are inactivating mutations in the antigen presenting components.

Loss of both copies of MHC I heavy chain genes or of ß2M will eliminate essentially all MHC I expression, and such loss does occur in cancers (96, 141, 158, 159). Loss of one copy of MHC I heavy chain or ß2M genes (loss of heterozygosity) also has been documented in many cancers (95, 143, 158, 160–165). In a survey of 59 cancer types, loss of MHC I heterozygosity was observed to occur in 17% of cancers (166). That this might be a consequence of immunoediting was suggested by the observation that this loss occurred more frequently in cancers with higher mutational burdens and therefore ones that were expressing potentially more immunogenic neoantigens (166). Because MHC I heavy chain genes are co-dominantly expressed from both chromosomes, loss of one copy of an MHC I heavy chain gene reduces MHC I expression by about 50% (35). In cells with MHC I loss of heterozygosity, a single inactivating mutation in a remaining MHC I gene will lead to a null phenotype and such mutations do occur in the coding regions of individual MHC I heavy chain genes (143, 167). Over time MHC I expression can decrease in patients, with e.g., primary lesions being MHC I positive but metastasis losing such expression, presumably the result of immunoediting (93, 168).

**Figure 6:**
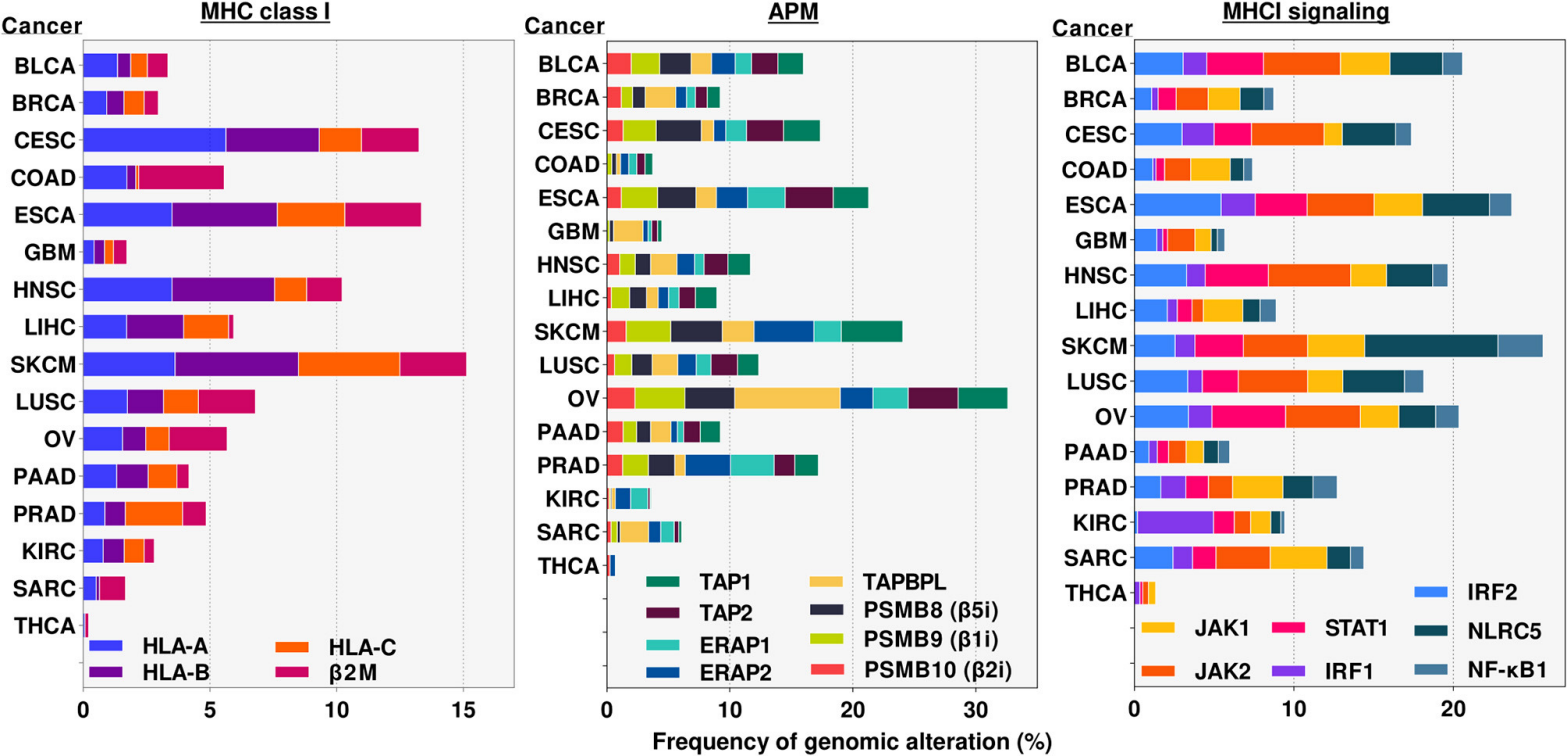
Frequency of alterations in genomic sequences of MHC class I pathway genes in various cancers. Genomic alterations include any mutation, deletion and/or amplification occurring in either intron or exon regions of the indicated genes. Results were obtained from cBioportal.org (169, 170). analyses of publicly available TCGA data sets for the indicated genes and cancers. APM, Antigen Presentation Machinery. Cancer abbreviation are as in Figure 4.

Mutations and deletions also occur in all of the other components of the MHC I antigen presentation and IFN pathways as shown in sequencing data of many human cancers (Figure 6) (171). Much of this data has not been analyzed to tell whether and how often these genetic alterations have led to a loss of function, nor how many of the various cancers are free of any mutation in an MHC I pathway component. However, there are a number of reports of inactivating mutations and deletions of several of these components (95).

## Loss OF the MHC I Antigen Presentation Pathway In Cancers Through Transcriptional Regulation

In many cancers with MHC I pathway defects, there is an underlying loss of transcription of MHC I pathway genes (172, 173). In an individual cancer, this process can affect the expression of multiple MHC I pathway genes at the same time, including MHC I heavy chains, ß2M, TAP, Tapasin, ERAP1, and immunoproteasome subunits (105, 173–176). The underlying mechanisms for such loss of MHC I pathway gene expression have been elucidated for some cancers.

One mechanism that affects transcription of MHC I pathway genes in cancers is a loss of key transcription factors. The NLRC5 transcription factor is reduced in multiple cancers including prostate, lung, uterine, melanoma, and thyroid cancers and this is correlated with a reduction in the expression of its target genes, including MHC I, ß2M, TAP, and immunoproteasome subunits (123) The loss of NLRC5 could arise from loss or mutation of the gene (Figure 6), or methylation of its promoter or associated histones (123, 177, 178). Loss of nuclear IRF1 in melanomas is associated with resistance to checkpoint blockade (179). Loss of expression of NF-κB and IRF1 in neuroblastomas results in a loss of MHC I expression (180). Loss of IRF2 caused a downregulation of many components of the MHC I antigen presentation pathway (MHC I heavy chains, immunoproteasomes, TAP, TAPBPR, and ERAP1) as well as an increase in CD274 (PDL-1) (69). Many human cancers (e.g., breast, NSCLC, prostate, colorectal, and uterine) downregulate IRF2 expression, which results in an immune evasion phenotype with MHC I low and PD-L1 high expression (69).

**Figure 7:**
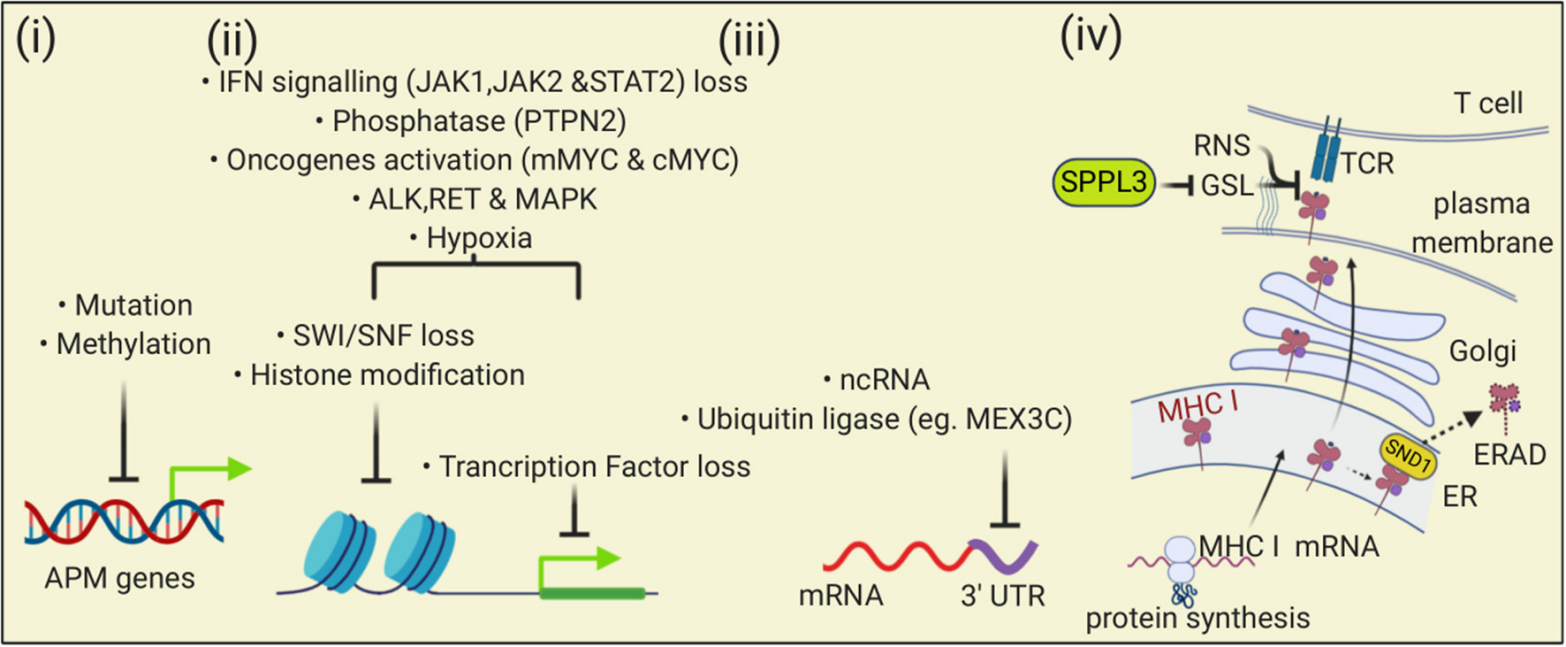
The MHC I antigen presentation pathway is down-regulated by multiple mechanisms in cancers. (i) At the level of DNA, mutation and methylation of nucleotides can reduce expression of APM genes; (ii) Transcription of APM can be reduced by changes in chromatin that impair gene accessibility or through loss of transcription factors. Multiple mechanisms can affect these processes including altered signaling pathways, oncogene activity, and the tumor microenvironment; (iii) At the level of transcription, binding of ncRNA or proteins to the 3′ UTR of APM mRNAs can reduce transcription; (iv) At the level of protein, Staphylococcal nuclease and tudor domain containing 1 (SND1) can bind to MHC I in the ER and trigger endoplasmic reticulum-associated degradation (ERAD). At the cell surface, loss of SPPL3 increases glycosphingolipids (GSL) that then sterically inhibit MHC I and TCR interactions. Reactive nitrogen species can nitrosylate peptide-MHC I complexes in ways that impair TCR interactions.

The expression of antigen presentation pathway genes can be downregulated through epigenetic silencing (Figures 2, 7). One such mechanism that has been observed in several cancer types is hypermethylation of the promoters or enhancers of these genes. This modification has been documented in the regulatory elements of MHC I (176, 181–183), TAP (128), Tapasin (184), IFNR pathway components (185–187). This DNA modification silences gene expression by recruiting repressive factors, such as methyl-CpG binding domain protein 1 (MBD1) and methyl-CpG binding protein 2 (MeCP2) and interfering with transcription. As cancer cells divide, these methylated sequences are passed onto daughter cells, thereby perpetuating the gene silencing. Treatment with agents that cause DNA demethylation has restored MHC I expression in some cancers, demonstrating the importance of this silencing mechanism (182, 188). Cancer neoantigen genes can also be subjected to DNA hypermethylation (189).

Another epigenetic silencing mechanism involves histone modifications, which are also heritable to daughter cells. Histone acetylation can alter chromatin in ways that increase DNA accessibility and thereby allowing entry and binding of transcription factors. Histone deacetylase (HDAC) inhibitors, which lead to increased acetylation levels, have restored expression of MHC I molecules and other antigen presentation components in some cancers, suggesting that histone deacetylation silences expression of MHC I pathway genes (190–194). Trimethylation of histones (e.g., H3K27me3) can also downregulate genes by affecting the state of heterochromatin. In some MHC I low cancers, H3K27me3 repressive marks are associated with the promoters of NLRC5, MHC I heavy chain genes, β2M, immunoproteasomes and TAP, and loss of this repressive modification results in an increase in MHC I pathway expression (177, 195).

The polycomb repressive complex 2 (PRC2) was found to be a repressor of MHC I expression in some cancer cells, such as neuroblastomas and small cell lung carcinomas (177). PRC2 silences the basal expression of NLRC2, MHC I, immunoproteasomes, and TAP and also inhibits IFNγ-induced MHC I upregulation (177). A subunit of the PRC2 complex binds and activates the lysine methyltransferase EZH2. Consistent with this mode of action, the repression caused by PRC2 was associated with increased H2K27me3 histone repressive marks associated with MHC I pathway genes, which when reversed, increased transcription factor binding and MHC I expression. Consistent with these results, deletion of EZH2 in leukemia cells increased MHC I expression (196) and activating mutations of EZH2 caused a loss of MHC I expression in these cancers (197).

SWI/SNF factors affect gene expression by regulating chromatin accessibility. The Polybromo-associated BAF (PBAF) SWI/SNF complexes were found to be a positive regulator of MHC I expression (198). The expression of PBAF in cancers is correlated with better prognosis and responsiveness to checkpoint blockade. Interestingly for PBAF, it particularly affects promoter accessibility of IRF2 and interferon-stimulated response elements (ISREs) (198).

## loss of the MHC I Antigen Presentation Pathway In Cancers Through Post-Transcriptional/Pre-Translational Regulation

The expression of proteins can be regulated through post-transcriptional mechanisms (Figure 7) and one of these mechanisms is mediated by non-coding RNAs (ncRNA) (199). One class of ncRNAs are small (22 bp average length) microRNAs (miRNA). These sequences can bind to the 3′ untranslated regions (UTR) of mRNAs and inhibit their translation through repression or by targeting them for degradation.

There are a number of examples of miRNAs that regulate the expression of components of the MHC I antigen presentation pathway and can contribute to a loss of antigen presentation in cancers (66, 200). In some cancers, there is increased expression miRNAs and it has been shown that overexpression of these miRNAs leads to a reduction in MHC I pathway components. For example, in esophageal cancer, miR-148a-3p was found to bind to untranslated regions (UTR) of MHC I transcripts and miR-125a-5p bound to UTRs of TAP2 transcripts. Moreover, the overexpression of these miRNAs reduced the expression of these antigen presentation components (201). In melanomas, miR-26b-5p and miR-21-3P bind the UTR of TAP1 transcripts and downregulate TAP1 expression (202). In colorectal cancers, miR-27a expression is increased and causes reduced MHC I expression by suppressing expression of calreticulin (203). Mir-502-5P in gastric cancer and miR-23a in hepatocellular cancer were found to reduce IRF1 expression (204, 205). Thus, miRNAs, which can be highly expressed in cancers, can negatively regulate many of the components of the MHC I antigen presentation pathway. Investigations in this subject area have been relatively limited and therefore it is likely that many more examples of miRNA-mediated inhibition of MHC I antigen presentation in cancers are yet to be discovered.

Another class of ncRNAs are long (>200 bp) non-coding RNAs (lncRNA). These sequences can regulate gene expression in many ways, including epigenetically, transcriptionally and post-transcriptionally (199, 206). One interesting example of a lncRNA that regulates MHC I antigen presentation post-transcriptionally is LINK-A. In a breast cancer model, LINK-A inhibited antigen presentation by indirectly stimulating an E3 ubiquitin ligase which led to the degradation of the peptide-loading complex (207). Again, it is highly likely that additional lncRNAs will be found to negatively regulate components of the MHC I antigen presentation pathway.

ncRNAs can also be positive regulators of MHC I. For example, in head and neck squamous cell carcinomas, expression of the lncRNA, lnc02195, increases MHC I expression and is associated with a better prognosis (208). In nasopharyngeal carcinomas miR9 expression increases expression of MHC I molecules and TAP1 (209). Whether down-regulation of these ncRNAs in cancers is an important mechanism for immune evasion remains to be determined.

The UTR regions of mRNAs can be regulated not only by ncRNAs, but also by proteins binding to these sequences. An RNA-binding E3 ubiquitin ligase, MEX-3C, binds to the 3′ UTR of the transcript for MHC class I molecule HLA-A2 leading to its degradation (210); whether this mechanism is operative in and important to cancer immune evasion has not yet been examined.

## Loss of the MHC I Antigen Presentation Pathway in Cancers Through Post-translational Mechanisms

There are post-translational mechanisms that can impair MHC I antigen presentation in cancers (Figure 7). One such post-translational mechanism is analogous to immune evasion mechanisms employed by some viral pathogens. Some viruses encode immune evasion molecules that cause MHC I complexes to be dislocated from the ER into the cytoplasm, where they are degraded through a process referred to as endoplasmic reticulum-associated degradation (ERAD). It turns out that the oncoprotein Staphylococcal nuclease and tudor domain containing 1 (SND1), which is highly expressed in a number of cancers (e.g., Prostate and Melanoma), binds MHC I molecules and causes them to undergo ERAD. Deletion of SND1 in some cancer cell lines increases MHC I expression (211).

Another interesting mechanism that inhibits MHC I antigen presentation is a change in glycolipids on the plasma membrane that occurs in cancer cells that lose the signal peptide peptidase-like 3 (SPPL3) protease (212). SPPL3 cleaves and inactivates a glycosyltransferases B3GNT5, and loss of B3GNT5 reduces levels of negatively charged glycosphingolipids (GSL). Loss of SPPL3 results in an increase in these GSLs, which then associate with MHC I molecules in ways that appear to sterically inhibit their interaction with T cells. This process occurs in gliomas (and potentially some other cancers) and impairs T cell responses to these cells.

Yet another interesting post-translational mechanism that interferes with MHC I antigen presentation is modification of amino acid residues in the peptide-binding groove of MHC I molecules that alters peptide binding; this mechanism is described in more detail in the next section.

## Loss of the MHC I Antigen Presentation Pathway In Cancers Due to Signaling Mechanisms and Extrinsic Stimuli From the Tumor Microenvironment

Alterations in signaling pathways can lead to MHC I downregulation in cancers (Figure 7). MAPKs, which are activated in some cancers, are negative regulators of MHC I (213, 214). Inhibiting or silencing of MAPKs increased levels of IRF1 and STAT1 (215) as well as MHC I expression (214). MAPK inhibitors increased mRNA expression of MHC I, TAP, and ß2M (214). Similarly, inhibition of the ALK and RET kinases, which are upstream activators of MAPK, also increase MHC I expression and interestingly also results in the presentation of a different repertoire of peptides (216). Another example is that signaling though the EGFR oncogene HER2/neu is associated with a loss of transcripts for immunoproteasome subunits and TAP, resulting in a loss of MHC I surface expression and antigen presentation (217, 218). Yet another example is that n-MYC and c-MYC overexpression caused loss of MHC I expression, potentially by affecting NF-κB (219–221).

IFNs that are present in the tumor microenvironment [e.g., IFNγ produced by activated T cells or type I interferons produced by a variety of cells, bind to interferon receptors (IFNR) on tumor cells]. Signaling through the IFNRs (Figure 2) leads to an increase in expression of many components of the MHC I antigen presentation pathway (e.g., MHC I, TAP, Tapasin, immunoproteasomes, and ERAP1). Components of the IFN pathway can also be lost (Figure 7) and this prevents IFN-induced upregulation of the MHC I pathway of antigen presentation. Such loss can also reduce basal levels of MHC I molecule expression (222, 223). IFN receptors signal through Janus kinases (Jak1 and Jak2) and STAT (STAT 1 and STAT2) proteins (224) (Figure 3). LOH and/or mutations in Jaks, and STATs are observed in cancers (143, 171). Loss of function mutations in Jak kinases with consequent loss of responsiveness to INFγ were found in Melanomas that became resistant to checkpoint blockade, pointing to the likely clinical significance of the inactivation of the IFN pathway (147, 151, 225). Loss of function in a receptor (APLNR) that interacts with Jak1, reduces IFNγ-stimulated Jak1 and STAT signaling and MHC I upregulation. Mutations in this receptor are found in melanoma patients that are resistant to checkpoint blockade and similarly such resistance is conferred upon knock out of this receptor from mouse melanoma cells (142). Loss of the tyrosine protein phosphatase Ptpn2 that represses IFNγ signaling by dephosphorylating both JAK1 and STAT1. Deletion of Ptpn2 in mouse tumors increases MHC I antigen presentation and improve immunotherapy (226); whether increased Ptpn2 activity in human tumors causes a loss of MHC I antigen presentation is not known. Finally, TGF-ß, which can be present in the tumor microenvironment, can cause a down-regulation of MHC I molecules in some cancers (e.g., ovarian, prostate, and ocular melanoma) (227–229).

Other events in the tumor microenvironment can lead to impaired MHC I antigen presentation in cancers (Figure 7). Tumor microenvironments can be hypoxic and hypoxia can impair MHC I antigen presentation in cancers, in part by inhibition of STAT1 (230). Tumor-infiltrating myeloid cells produce reactive nitrogen species in the tumor microenvironment, and this can impair MHC I antigen presentation in cancers. In this situation, the reactive nitrogen species cause nitrosylation of residues in the MHC I peptide binding site, which can inhibit the binding of peptides (231). In tumor-bearing mice, myeloid suppressor cells cause defective IFN responses in host cells (responses in tumors were not examined) likely due to a STAT1 defect potentially caused by nitrosylation (232).

## Gene Disruptions That Affect MHC I Antigen Presentation: Evidence From Forward Genetic Screens In Cancers

Recently, a number of forward genetic screens have been performed in cancer cells subjected to selection for decreased or increased MHC I expression and have identified a large number of new gene candidates that are potentially involved in MHC class I antigen presentation (69, 142, 167, 174, 177, 226, 233–235). In fact, several of the genes described above (IRF2, PBAF, PRC2, and SPPL3) were discovered in such screens. It is important to note that many of the gene candidates that are initially identified may be artifacts. Therefore, all candidates require further validation and analyses to determine whether they are affecting the MHC I antigen presentation pathway and involved in cancer immune evasion.

A recent CRISPR-cas9 screen in B cell lymphoma cell lines did repeat gene disruptions for individual candidates and were able to reproduce a loss or increase in MHC I expression upon disruption of ~200 genes (196). Among these genes were ones that are thought to be involved in endocytosis and vesicular trafficking, ubiquitin conjugation, ER quality control, as well as other processes. Further work is needed on these and candidates from other screens to determine whether they are involved in cancer immune evasion. However, interestingly, 30 of these genes showed correlations with CD8 T cell infiltration in multiple cancers; 10 negative-regulatory genes were correlated with less tumor infiltrating CD8 T cells and 20 positive-regulatory were correlated with more infiltrating CD8 T cells. The field can look forward to much more information on the role of these genes and other validated ones in MHC I antigen presentation and cancer immune evasion.

## Potential for Restoring MHC I Expression In Cancers

The fact that the loss of MHC I antigen presentation is common in cancers and allows these cells to evade immune surveillance, raises the question of whether the MHC I pathway defects could be reversed so as to reestablish immune control and responses to immunotherapy. For cancers with deletions or inactivating mutations in structural antigen presenting genes, this would require gene replacement or editing in most cases. *In vitro*, this has been successfully accomplished by transfection of MHC I pathway genes into cancer cell lines. Similarly, gene therapy with a ß2M-adeno-viral vector has been successful in restoring MHC I expression *in vivo* in a murine model (236–238). However, for gene transfer or editing to be a viable therapy, it will likely require that all cancer cells (in the primary site and metastasis) to be transduced and “repaired,” because otherwise MHC I-low clones would continue to grow. Achieving this level of gene expression or repair is probably not feasible with current gene therapy technology. An exception for overcoming the loss of structural genes is the situation where the function of the lost gene can be replaced by inducing another functionally redundant gene. One example of this is where MHC I expression in cancer cells was lost by deletion of the IRF2 transcription factor, but then restored by inducing IRF1 with IFN-stimulation (69). In this case IRF1 and IRF2 are both activating transcription factors that bind to the same promotor element (239).

In situations where the MHC I pathway structural genes are intact but their expression is downregulated, there is the potential to restore gene expression. In some MHC I low cancers, treatment with IFNs has increased MHC I levels (120, 240, 241). The mechanism as to how IFN is restoring MHC I expression has not been investigated in detail, except in one study, IFN was shown to cause increased histone acetylation, DNA demethylation of the promoters of TAP and immunoproteasome genes, and increased transcription of these and other antigen presenting genes (241). In addition, it is possible that IFN is also increasing MHC I levels in MHC I-low cancers through induction of IRF1, which then drives more transcription of the MHC I pathway genes (69), but this has not generally been examined. There are recombinant type I and II IFNs that work *in vivo* and are FDA-approved for other indications. In a small phase 2 trial in which two patients had MHC I negative melanomas, systemic IFNγ-administration induced MHC I expression (240). IFNγ has been shown to improve outcomes of checkpoint blockade in one clinical trial in melanoma (242), however whether and how much this had to do with MHC I expression is unknown.

For cancers that have lost MHC I expression due to epigenetic silencing mechanisms, there may be the potential to restore MHC I expression by reversing the repressive epigenetic marks. There are several examples where MHC I low cancer cell lines have been treated with drugs that inhibit DNA methyltransferases, the enzymes that are responsible for methylating DNA (181, 182, 243, 244), and thereby reverse gene silencing, presumably through demethylation of promoters. Such treatment has increased MHC I expression in several MHC I low cancers cell lines (181, 182, 188, 245). Where examined, this class of epigenetic modifying drugs was found to restore MHC I expression by upregulating expression of many IFN-responsive gene (246) including MHC I antigen presentation pathway genes in cell lines (95, 241, 245). These findings raised the possibility that this class of agents could augment T cell-based immunotherapy. Consistent with this idea, this class of agents has been shown to augment or give additive effects with checkpoint inhibitors in preclinical mouse models (182, 247, 248). There are several DNA methyltransferase inhibitors that are approved by the FDA for cancer treatment, although the exact basis for their efficacy (i.e., what are the key pathways that are affected to give the anti-cancer effects, isn't known). These drugs have been shown to increase expression of IFN and MHC I antigen presentation pathway genes in cancers *in vivo* (182, 245) and in limited clinical trials have improved responses to checkpoint blockade immunotherapy (249) and a tumor vaccine (250). Currently there are further ongoing trials of these agents in combination with immunotherapy (251). Inhibitors of the EZH2 methyltransferase, which as described above is a negative regulator of MHC I antigen presentation, can restore MHC I levels in lymphomas (196, 197).

Similarly, a number of MHC I low cancer cell lines have been treated with histone deacetylase (HDAC) inhibitors, which by increasing histone acetylation can restore promoter activity. HDAC inhibitors have also increased MHC I expression and MHC I pathway components in cancer cell lines (182, 190, 192, 193, 243, 252, 253). There are FDA-approved HDAC and DNA methyltransferase inhibitors that are used to treat cancers. Where studied *in vivo*, methyltransferase inhibitors increased expression of MHC I and MHC I pathway components in multiple types of cancers in patients (182, 245) and a xenograft model (192). In preclinical models, HDAC inhibitors and anti-PDL1 antibody (186, 193, 254–257) or with T cell therapy (258) gave additive effects. Combinations of HDAC inhibitors and checkpoint blockade have and continue to be tested in clinical trials (194, 259–261).

Based on the data just discussed, it is clear that epigenetic modifying agents can increase the MHC I antigen presentation pathway in some MHC I low cancers and that these drugs can improve responses to immunotherapy, however whether these two observations are causally related is not yet established. This is because epigenetic modifying drugs effect the promoter landscapes in potentially all cells. Therefore, these agents can affect not only the tumor, but also cells within the tumor microenvironment and immune system. Moreover, the drugs can affect the expression of many genes within these cells. In most studies, which of the key gene regulatory events that are responsible for therapeutic effects of these drugs is not known. Interestingly, in one animal study the therapeutic effect of an HDAC inhibitor was lost in immunodeficient mice (191), providing evidence that the drug was acting to improve immune control a cancer; however, whether this effect is *via* the restoration of the MHC I pathway or some other immune mechanism is not known.

The broad effects of the epigenetic modifying agents lead to multiple and sometimes opposing effects. For example, global DNA hypomethylation may increase MHC I expression, but also upregulate immunosuppressive mechanisms such as suppressive cytokines and checkpoint inhibitors (262, 263). Such complexity might be overcome, and outcomes improved if there were ways to more selectively modify epigenetic marks of particular genes. At present, drugs that inhibit individual HDAC enzymes are available, and perhaps even more selective agents will be developed. New gene editing approaches using modified Cas9 fusion proteins (e.g., Cas9-p300 acetyltransferase, Cas9-methyltransferases can Cas9-demethylases) have the ability to alter epigenetic marks and/or transcriptionally activate or repress expression of specific genes (264, 265). Whether such approaches could somehow be used *in vivo* to efficiently edit all cancer cells remains to be seen.

MicroRNAs that reduce MHC I antigen presentation are a potential therapeutic target. MiRNAs can be blocked in cells by treatment with complementary anti-sense RNAs (antimirRs/antagomiRs) and overexpression of miRNAs can be achieved using miRNA duplexes (miRNA mimetics) (199, 266). These approaches require chemical modifications to stabilize RNA oligonucleotides and methods to deliver these compounds into cells (e.g., lipid nanoparticles). Such agents have been used successfully in preclinical models and have and/or are being tested in phase 1 and 2 clinical trials, but not yet for affecting MHC I antigen presentation. LncRNAs can be targeted for degradation with anti-sense oligonucleotides.

Inhibitors of enzymes that lead to a loss of MHC I antigen presentation also have the potential to restore antigen presentation in some cancers. Inhibitors of ALK, RET, and MAPK kinases can reverse the downregulation of the MHC I antigen presentation caused by these enzymes (213, 214, 216). There are FDA approved inhibitors of glycosyltransferases, and these agents were shown to reverse the suppression of MHC I stimulatory activity caused by the increase in negatively charged glycosphingolipids from loss of SPPL3 (212). Inhibitors of thymidylate synthetase were found to increase MHC I levels in lymphoma cells in a targeted small molecule screen, *via* an as yet unknown mechanism (196).

Finally, in cells that have lost some MHC I antigen presentation pathway components, such as TAP (40, 267) and ERAP1 (60, 100), novel peptides peptides [aka “T cell epitopes associated with impaired **peptide** processing (**TEIPP)]** are presented that are not displayed in wild type cells and can be immunogenic. There is limited data that immunization with such sequences can elicit anti-tumor responses (268, 269). Therefore, an alternate approach to restoring the loss of MHC I antigen presentation components could be to exploit the presentation of unique antigen peptides that are displayed on such cancer cells (269).

The elucidation of the many mechanisms that lead to a loss of MHC I antigen presentation and the identification of tractable therapeutic targets to reverse this loss, brings the hope of restoring immune control and improving T cell-based immunotherapy. Given the differences between different cancer types, the heterogeneity within a single type of cancer, and the many different mechanisms that can disable the MHC class I pathway, it seems likely that such approaches will require precision medicine, where the cause of immune evasion in an individual patient is identified and then the appropriate therapy selected. The advances in identifying the underlying mechanisms that cripple MHC I antigen presentation are necessary steps in attempting to achieve this goal.

## Recognition and Control OF MHC I Low Cancers by NK Cells

Cancers that have lost MHC I expression through the mechanisms discussed above, can avoid control and elimination by CD8 T lymphocytes. This is analogous to the situation where some viruses encode immune evasion molecules that inhibit MHC I antigen presentation and thereby allow virally infected cells to avoid being killed by CD8 T cells and help establish chronic infections. In these situations, there is a second line of defense that can kill these abnormal cells and this immune function is provided by natural killer (NK) cells. NK cells identify these cells in part by sensing the presence or absence of MHC I molecules.

NK cells are a lineage of lymphocyte that is distinct from B and T cells. These cells have similar effector functions (cytotoxicity and cytokine production), as CD8 T cells. However, NK cells are innate lymphoid cells (ILC) and the receptors they use to recognize their target cells are fundamentally different from the ones used by T (and B) lymphocytes. Instead of employing a mechanism that recombines gene segments to generate clonally unique and diverse receptors, NK cells use non-rearranging germ-line encoded receptors of several different types (270). Some of these NK receptors are activating ones and others provide inhibitory signals.

Human NK cells express several killer inhibitory receptors (KIR) that upon engagement of their ligands, impart inhibitory signals through ITIM motifs in the receptors' intracytoplasmic domains. HLA-A and HLA-B are ligands for KIR3D receptors and HLA-C is recognized by KIR2D receptors. In addition, NK cells express other types of inhibitory receptors that recognize MHC I molecules, including CD94-NKG2A, which recognizes HLA-E, and LILRB1, which recognizes all MHC I molecules. Moreover, NK cells express other inhibitory receptors that are not involved in MHC I recognition (270). Because of their inhibitory receptors that recognize MHC I molecules, NK cells ignore normal MHC I-sufficient cells but are disinhibited when they encounter abnormal-MHC I low cells. This loss of inhibition is a necessary but not a sufficient event to trigger the NK cell's effector mechanisms.

Activation of NK cells requires engagement of activating receptors, which in humans include NKG2D, NKp20, NKp44, and NKp46 (270). These receptors associate with and signal through ITAM-containing proteins (270). Other stimulatory receptors expressed by NK include 2B4 (CD244) and DNAM1 (CD226) (270).

The ligands of some of the activating receptors are ones whose expression is induced on cancers, virally infected and stressed cells. The best characterized examples are the MHC class I polypeptide–related sequence A and B (MICA and MICB) molecules, which are the ligands of the activating NKG2D receptor. MIC A and MIC B are structurally similar to HLA class heavy chains but are not associated with ß2M and do not bind peptides. Because of these properties, MICA's and MICB's expression is not affected by defects in the MHC I antigen presentation pathway and therefore can be expressed in MHC I negative cancer cells. The activation of NK cells depends on the balance of stimulatory and inhibitor signals they receive. Cancers or virally infected cells that both express activating ligands and lack inhibitory ligands can be killed by NK cells.

Mice that lack functional NKs cell due to antibody treatment or genetic knock outs develop a higher frequency of some cancers (271, 272). Similarly, humans that have NK cell defects have increases in some cancers, particularly ones that may be induced by viruses (273). A caveat in many of these studies is that the mouse models and NK deficient humans may have defects beyond just a loss of NK cells (273). Nevertheless, the data in aggregate suggest that NK cells play a role in immune surveillance. Whether the protection against carcinogenesis afforded by NK cells is primarily against MHC I low cancers is not clear (274).

NK cells may still exert some control after cancers have arisen. Depletion of NK cells in mouse models promotes the growth and metastasis of transplanted tumors (271, 275, 276). Moreover, adoptive transfer of NK cells into tumor-bearing mice can lead to tumor rejection. In humans, the level of cytolytic activity of circulating NK cells correlates with the risk of cancer (277) and infiltration of NK cells in some cancers is associated with a better prognosis (278, 279). Similarly, adoptive transfer of NK cells into human cancer patients has shown therapeutic effects in some patients, demonstrating that similar principles likely apply in humans (280). Such studies have led to considerable interest in exploiting NK cells for tumor therapy and there are many efforts underway to do so [e.g., developing CAR-NK cell therapy and antibodies that recruit activated NK cells to cancers (270, 281, 282)].

Despite NK cells being a potential second line of defense against tumors that have lost MHC I, once such tumors become clinically evident, they almost always progress. In fact, as noted above, loss of MHC I is often a negative prognostic indicator. Moreover, there is no evidence that MHC I negative cancers are infiltrated with more NK cells than MHC I sufficient cancers (283). Therefore, for many MHC I low cancers, either they were never targets of NK cells or such tumors have evolved ways to evade control by NK cells.

In fact, there are many mechanisms through which cancers can evade NK cells. For example, HLA A, B&C low cancers can express the non-classical MHC I molecules HLA-G and HLA-E, which can inhibit NK cells by engaging their inhibitory receptors (284–288). In addition, NK cells may not penetrate into solid tumors or once within the cancer can become anergic or exhausted, including in MHC I low tumors (289–291). Furthermore, tumors can shed their MIC molecules, thereby removing an activation signal and creating a soluble ligand that can block the NK cell's cognate receptor (292, 293). Moreover, cancers can also create an immunosuppressive environment [e.g., producing TGFß, which can lead to inhibition of NK cells (294)]. A fuller consideration of these mechanisms is beyond the scope of this article and readers are referred to recent reviews (270, 295, 296).

## Future Directions

While there is abundant evidence that loss of MHC I antigen presentation is a frequent event in cancers that results in immune evasion, we still have much to learn. As reviewed above, new mechanisms for MHC I downregulation have recently been discovered and there will be more to be uncovered. Forward genetic screens are identifying new components that contribute to the MHC I pathway and it will be of importance to investigate how they contribute to MHC I phenotypes in cancers. Even among the known mechanisms for loss of antigen presentation, a majority of the analyses have been performed in limited cancer types and a more comprehensive understanding across more types of cancer is needed. Moreover, many of the underlying mechanisms for MHC I pathway loss need to be elucidated at higher resolution (e.g., causes and specific targets of epigenetic modifications).

Given the importance of MHC I antigen presentation for the immune control and immunotherapy of cancers, there is a need to develop therapeutic approaches to restore the MHC I pathway in cancers and this should be feasible in at least some cancers. This might itself be an immunotherapy by allowing a restoration of immune control. It might also provide adjunct therapy that could improve the percentage of patients that respond to immunotherapies and potentially extend the efficacy of immunotherapies to cancers that have been largely resistant to such therapy. The various mechanisms that cause MHC I pathway-loss might also serve as biomarkers to help identify patients that have the potential to respond, or not, to immunotherapy and/or have the potential for the loss of MHC I to be reversed. The hope for such biomarkers is that they could make immunotherapy more personalized (e.g., sparing those patients who won't respond to such therapy from the risks and enormous expense of the treatment).

## Conclusions

A sizable percentage of many different types of cancers lose MHC I antigen presentation partially or completely. This is almost certainly the result of immunoediting where MHC I low variants emerge under selection pressure imposed by CD8 T cells. The result of this process is that CD8 T cells can no longer “see” these MHC I-deficient variants and are therefore unable to control or eliminate them. This process reflects the fact that the MHC I pathway is non-essential for viability and growth and therefore when lost does not compromise cancer progression. Where examined, this escape of immune control is generally associated with worse prognoses and resistance to immunotherapy. There are many mechanisms that underlie the loss of MHC I antigen presentation. Some mechanisms involve mutations and deletions of structural genes of one or more component(s) of the antigen presentation pathway; others effect transcription of pathway genes *via* loss of transcription factors or epigenetic silencing of gene regulatory elements; and yet others can affect the stability of mRNAs for MHC I pathway components or the molecules themselves, or signaling pathways that regulate MHC I expression. Some of these alterations are unique to an individual cancer and others are common in multiple patients and cancer types. It will be important to further understand the multiple mechanisms for loss of the MHC I pathway that are operative in all cancer types and their clinical significance. The hope is that in the future, characterizing MHC I pathway lesions in individual patient samples would lead to actionable information about what therapies will or will not be likely to work and prognosis. Moreover, some of the mechanisms that cause the loss of the MHC I pathway in cancers are reversible and may be amenable to the development of therapeutic interventions that could make T cell-based immunotherapies more efficacious in more patients and in more kinds of cancer.

## Author Contributions

The text was written by KR, KD, and JC. Figures were made by KD and JC. All authors contributed to the article and approved the submitted version.

## Conflict of Interest

The authors declare that the research was conducted in the absence of any commercial or financial relationships that could be construed as a potential conflict of interest.

## Glossary

APM: Antigen presentation machinery
ß2M: Beta2 microglobulin
CTLA-4: Cytotoxic T-lymphocyte-associated protein
ER: Endoplasmic reticulum
ERAD: Endoplasmic-reticulum-associated protein degradation
ERAP1: Endoplasmic reticulum aminopeptidase 1
EZH2: Enhancer of zeste 2 polycomb repressive complex 2
GSL: Glycosphingolipid
HDAC: Histone deacyetylase
HLA: Human leukocyte antigen
ILC: Innate lymphoid cells
JAK: Janus kinase
KIR: Killer inhibitory receptor
lnkRNA: Long noncoding RNA
NK: Natural killer
NLRC5: NLR Family CARD domain Containing 5
MHC: Major histocompatibility complex
miRNA: microRNA
PD1/PD-L1: Programmed cell death protein 1/ Programmed death-ligand 1
TAP: Transporter of antigen presentation
TIL: Tumor infiltrating lymphocytes
UTR: Untranslated region.
